# Noise in a Metabolic Pathway Leads to Persister Formation in Mycobacterium tuberculosis

**DOI:** 10.1128/spectrum.02948-22

**Published:** 2022-10-04

**Authors:** Jeffrey Quigley, Kim Lewis

**Affiliations:** a Antimicrobial Discovery Center, Department of Biology, Northeastern University, Boston, Massachusetts, USA; National Center for Biological Sciences

**Keywords:** *Mycobacterium tuberculosis*, antibiotic tolerance, persisters

## Abstract

Tuberculosis is difficult to treat due to dormant cells formed in response to immune stress and stochastically formed persisters, both of which are tolerant of antibiotics. Bactericidal antibiotics kill by corrupting their energy-dependent targets. We reasoned that stochastic variation, or noise, in the expression of an energy-generating component will produce rare persister cells. In sorted M. tuberculosis cells grown on acetate, there is considerable cell-to-cell variation in the level of mRNA coding for AckA, the acetate kinase. Quenching the noise by overexpressing *ackA* sharply decreases persisters, showing that it acts as the main persister gene under these conditions. This demonstrates that a low energy mechanism is responsible for the formation of M. tuberculosis persisters. Entrance into a low-energy state driven by noise in expression of energy-producing enzymes is likely a general mechanism by which bacteria produce persisters.

**IMPORTANCE** M. tuberculosis infection requires the administration of multiple antibiotics for a prolonged period of time. Treatment difficulty is generally attributed to M. tuberculosis entrance into a nonreplicative, antibiotic-tolerant state. M. tuberculosis enters this nonreplicative state in response to immune stress. However, a small population of cells enter a nonreplicative, multidrug-tolerant state under normal growth conditions, absent any stress. These cells are termed persisters. The mechanisms by which persisters enter a nonreplicative state are largely unknown. Here, we show that, as with other bacteria, M. tuberculosis persisters are low-energy cells formed stochastically during normal growth. Additionally, we identify the natural variation in the expression of energy producing genes as a source of the stochastic entrance of M. tuberculosis into the low-energy persister state. These findings have important implications for understanding the heterogeneous nature of M. tuberculosis infection and will aid in designing better treatment regimens against this important human pathogen.

## INTRODUCTION

Mycobacterium tuberculosis infection is the leading cause of mortality as a result of an infectious disease, causing 1.4 million deaths per year (1). This ongoing global epidemic stems from the difficulty of eradicating the pathogen with currently available antibiotics. Treatment of antibiotic-susceptible M. tuberculosis requires 6 months with a combination of rifampicin, isoniazid, ethambutol, and pyrazinamide (2). Not surprisingly, this results in side effects and poor compliance. The need for a lengthy treatment is attributed to the presence of dormant, nonreplicating M. tuberculosis cells that are tolerant of killing by antibiotics (3–5).

The disease is typically associated with the walling off of M. tuberculosis in a granuloma, a complex structure made primarily of immune cells and their products (6). The various stressors in this environment induce a population-wide low-metabolism state termed dormancy. DosR and PhoP regulators control entrance of cells into a nonreplicative state under hypoxia and acid stress (7–10), and RelA mediates starvation-induced nonreplication (11, 12). Regardless of the stress, induction of dormancy is characterized by a metabolic downshift in the population and increased antibiotic tolerance (13–15). M. tuberculosis organisms incapable of this metabolic downshift are more susceptible to antibiotics *in vivo* (16).

Apart from this population-wide response to distinct stress factors, M. tuberculosis also forms a small subpopulation of persister cells that are produced stochastically during normal growth and are tolerant of killing by antibiotics (17–20). Persisters were originally discovered by Bigger and by Hobby et al. in the 1940s (21, 22), and decades later, they are attracting increased interest due to their role in recalcitrance of chronic diseases to antibiotic therapy (23). Tolerance is likely based on a shared feature of bactericidal antibiotics—killing by corrupting their targets (24). For example, aminoglycosides such as streptomycin cause mistranslation, which leads to the production of toxic misfolded peptides (25). Based on this, we suggested that persisters are low-energy cells (26). Indeed, persisters in Staphylococcus aureus and Escherichia coli have low levels of ATP (26–28). Sorting of cells treated with antibiotics with low levels of expression of tricarboxylic acid (TCA) cycle enzymes enriches for persisters in these two species. However, the relative input of these enzymes into persister formation is unknown, and detection of both expression of an energy-producing component and ATP in the same cell has not been achieved yet. The mechanism by which multidrug-tolerant M. tuberculosis persisters are formed is largely unknown. Given that both cells undergoing population-wide dormancy and persisters that can form during growth exhibit antibiotic tolerance, understanding the mechanism of M. tuberculosis persister formation is critical for developing more effective therapies to treat this important disease.

Here, using single-cell analysis, we demonstrate that M. tuberculosis persisters are stochastically generated low-ATP cells. It is this low-energy state that renders them tolerant of antibiotics. Further, using direct measurement of ATP and transcription in metabolic enzymes in the same cell, we explored the mechanistic basis of persister formation in M. tuberculosis. We show that, in a simple growth medium with acetate, low-ATP cells express low levels of the acetate kinase AckA. By overexpression of AckA, we were able to dramatically reduce the level of persisters, indicating that AckA functions as the main persister gene under these conditions. The approaches described in this study provide a means to determine the relative contribution of any gene into persister formation. Stochastic entrance into a low-ATP state is likely a general mechanism of persister formation in bacteria.

## RESULTS

### Low levels of ATP are linked to antibiotic tolerance in M. tuberculosis.

**FIG 1:**
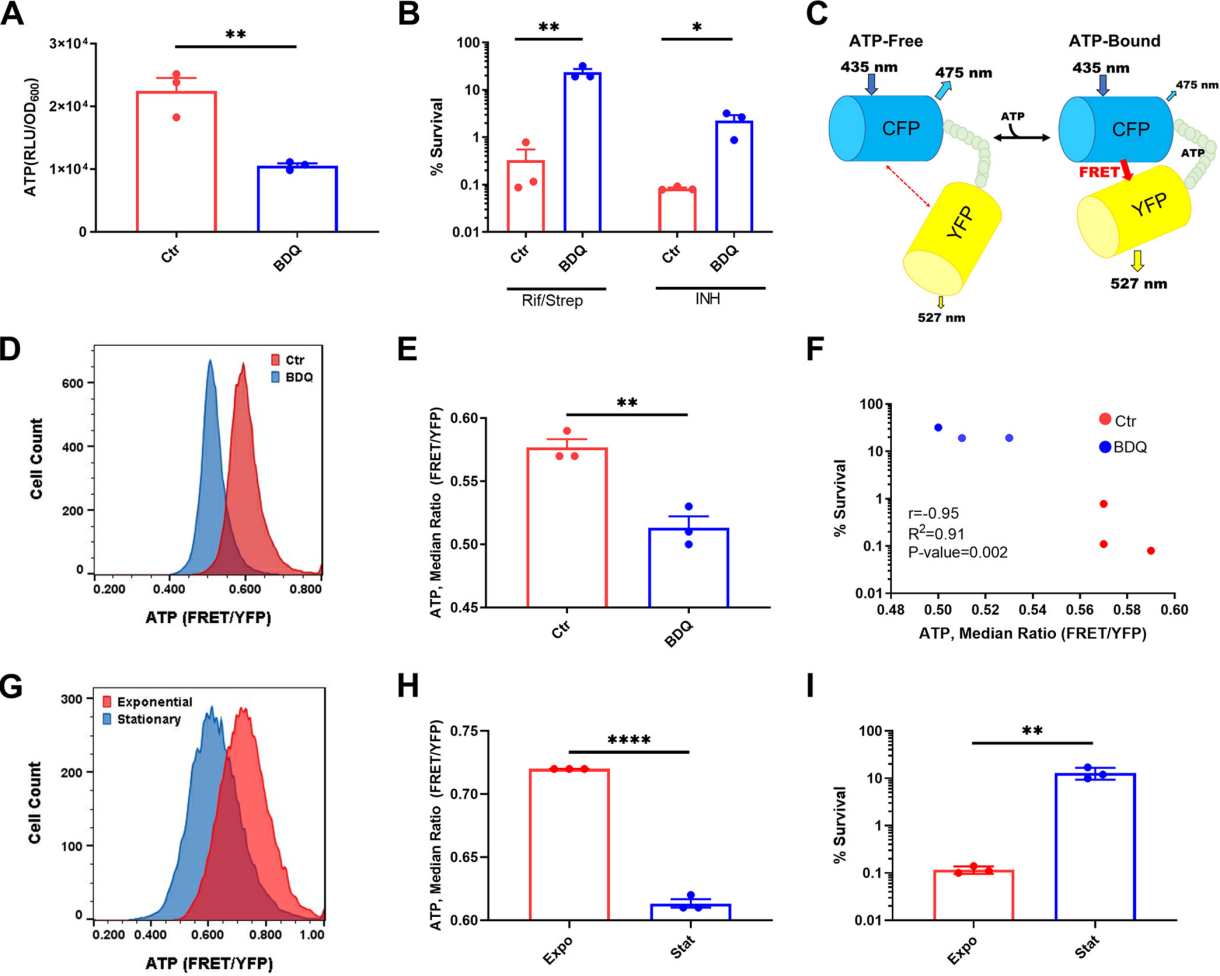
ATP level determines persister formation. Cultures of M. tuberculosis were grown in minimal medium with 5 mM acetate and treated with bedaquiline (BDQ) (12.5 μg/mL) for 4 h (A to F) or in 7H9 rich medium (G to I). (A) Luminescence-based ATP measurement after treatment with BDQ. Data are displayed as relative light units (RLU) normalized to OD_600_ of the culture. (B) Survival of BDQ treated cells after a challenge with either rifampicin (10 μg/mL) plus streptomycin (10 μg/mL) or isoniazid (20 μg/mL) for 7 days. The number of CFU per milliliter was determined at days 0 and 7. (C) Schematic of the ratiometric FRET-based ATP biosensor ATeam1.03^YEMK^. (D) Representative flow cytometry analysis of M. tuberculosis expressing ATeam1.03^YEMK^ and treated with BDQ (12.5 μg/mL) for 4 h. Data are displayed as FRET signal normalized to reporter expression (YFP). (E) Quantification of flow cytometry analysis in panel D. Data are displayed as the median FRET/YFP ratio. (F) Correlation analysis of percent survival and median FRET/YFP ratio of untreated and BDQ-treated M. tuberculosis cells. Data are representative of at least 3 biological replicates. Ctr, control (untreated cultures). (G) Representative example of single-cell ATP analysis via ATeam1.03^YEMK^ of exponential- and stationary-phase M. tuberculosis cells. (H) Quantification of medium FRET/YFP ratio in panel G. (I) Survival of exponential- and stationary-phase cells after treatment with rifampicin (10 μg/mL) plus streptomycin (10 μg/mL) for 7 days. The number of CFU per milliliter was determined before antibiotic addition and after 7 days of treatment. *, *P* < 0.05; **, *P* < 0.01; ****, *P* < 0.0001. Data are representative of three biological replicates. Significance was determined by unpaired two-tailed *t* test.

In order to examine a causal link between a low-energy state and persister formation in M. tuberculosis, we took advantage of the antibiotic bedaquiline, which specifically inhibits the mycobacterial F_1_F_o_ ATP synthase by binding to its C subunit (29). Bedaquiline is a bactericidal antibiotic but requires several days to kill M. tuberculosis. We found that adding bedaquiline at a high concentration (12.5 μg/mL, 100× MIC) to an exponentially growing culture for a short period of time, 4 h, did not affect viability. This treatment reduced the level of ATP 2-fold, as detected with luciferase (Fig. 1A). In order to measure the level of persisters in these cells, cultures were washed to remove bedaquiline and challenged with either rifampicin plus streptomycin (Rif/Strep) or isoniazid (INH) for 7 days, and viability was determined by colony count. Because M. tuberculosis readily develops resistance to rifampicin, for bulk persister assays, rifampicin was used in combination with streptomycin. After treatment with a variety of bactericidal antibiotics for 7 days, the bulk of the population is killed, and only persisters survive (17). Pretreatment with bedaquiline significantly increased the number of antibiotic-tolerant persister cells when challenged with either Rif/Strep or INH (Fig. 1B). Rifampicin inhibits RNA polymerase, streptomycin causes mistranslation of the ribosome, and INH is a prodrug that forms an adduct with NAD, which inhibits the synthesis of mycolic acid of the mycobacterial cell wall. Tolerance of these mechanistically unrelated antibiotics shows that a decrease in ATP causes multidrug tolerance. A longer (3-day) treatment with bedaquiline kills M. tuberculosis (30), apparently lowering the concentration of ATP to a point of no return. However, the ability of bedaquiline to cause multidrug tolerance of the pathogen is a potential cause for concern and should be taken into account when developing treatment regimens.

In order to analyze ATP in single cells, we employed ATeam1.03^YEMK^, a biosensor developed for use in mycobacteria (31). ATeam1.03^YEMK^ is a FRET (fluorescence resonance energy transfer)-based sensor comprising a pair of cyan and yellow fluorescent proteins (CFP and YFP) flanking the epsilon subunit of the Bacillus subtilis F_o_F_1_ ATP synthase, which binds ATP with high affinity and specificity (31). Binding of ATP by the epsilon subunit brings CFP in close proximity to YFP, resulting in energy transfer between the fluorescent proteins. To determine ATP concentration, FRET fluorescence is monitored using a setting optimized for excitation of CFP and emission of YFP (Fig. 1C). CFP is excited at 435 nm and emission is monitored at 527 nm (YFP), CFP_ex_→YFP_em_. FRET-based energy transfer from CFP to YFP is dependent on ATP concentration. The FRET values are normalized to fluorescence measurement of YFP by excitation at 488 nm and collecting emission at 527 nm, YFP_ex_→YFP_em_, which is not dependent on ATP concentration. This allows normalization of cell-to-cell variation in the levels of the reporter. Normalized values are displayed as FRET/YFP and are indicative of intracellular ATP concentration. ATP was monitored in single cells of a growing culture of M. tuberculosis mc^2^6020 expressing ATeam1.03^YEMK^ by fluorescence-activated cell sorting (FACS). Treatment with bedaquiline produces a distinct shift to lower levels of ATP (Fig. 1D to F). Next, we compared ATP levels in single cells of growing and stationary cultures. As expected, ATP levels are higher in a growing population (Fig. 1G and H). The level of persisters surviving treatment with Rif/Strep was 50-fold higher in the stationary population than in growing cells (Fig. 1I), in agreement with previous findings (19).

**FIG 2:**
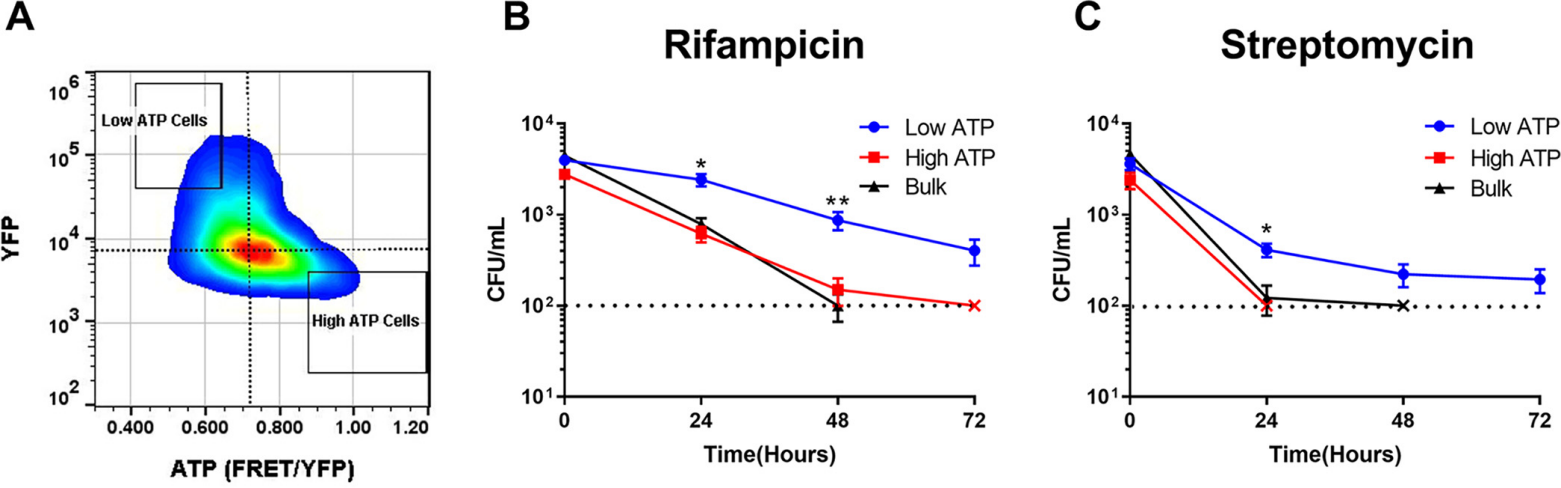
Low-ATP M. tuberculosis cells are multidrug tolerant. (A) Gating strategy for sorting low- and high-ATP M. tuberculosis from a population expressing ATeam1.03^YEMK^. Low- and high-ATP gates represent 2% of the total population. (B and C) Five thousand low-ATP, high-ATP, or bulk population cells were sorted directly into 7H9 medium containing (B) rifampicin (1 μg/mL) or (C) streptomycin (2 μg/mL). Survival was monitored by determining the number of CFU per milliliter at time zero and at 24, 48, and 72 h. Dotted lines represent the limit of detection. The symbol “×” indicates the time point at which a population fell below the limit of detection. *, *P* < 0.05; **, *P* < 0.01. Data are representative of at least 3 biological replicates. Significance was determined by multiple unpaired *t* test.

We took advantage of a higher level of persisters in the stationary population to directly examine the relationship between ATP and survival in single cells using cell sorting. A gate corresponding to 2% of the population was set to sort 5,000 low- or high-ATP cells (Fig. 2A) directly into a medium with either rifampicin or streptomycin, and survival was monitored for 72 h. The antibiotics were used individually, as the low number of cells sorted made generation of resistance to rifampicin unlikely. Additionally, 5,000 cells were sorted irrespective of the ATP state of the cell (YFP only) and were considered representative of the behavior of the bulk culture. The gate was set for low-ATP cells with good expression of the sensor (high YFP signal) in order to improve detection and avoid defective or dead cells. Low-ATP cells were considerably more tolerant of rifampicin (Fig. 2B) or streptomycin (Fig. 2C) than high-ATP cells or the bulk of the population. High-ATP and regular cells were essentially eliminated by rifampicin by 48 h, while a distinct population of low-ATP cells survived at 72 h. A similar pattern was observed with a more rapidly killing streptomycin. This experiment shows that low-ATP cells produced stochastically are multidrug tolerant.

### Identifying noise generators.

**FIG 3:**
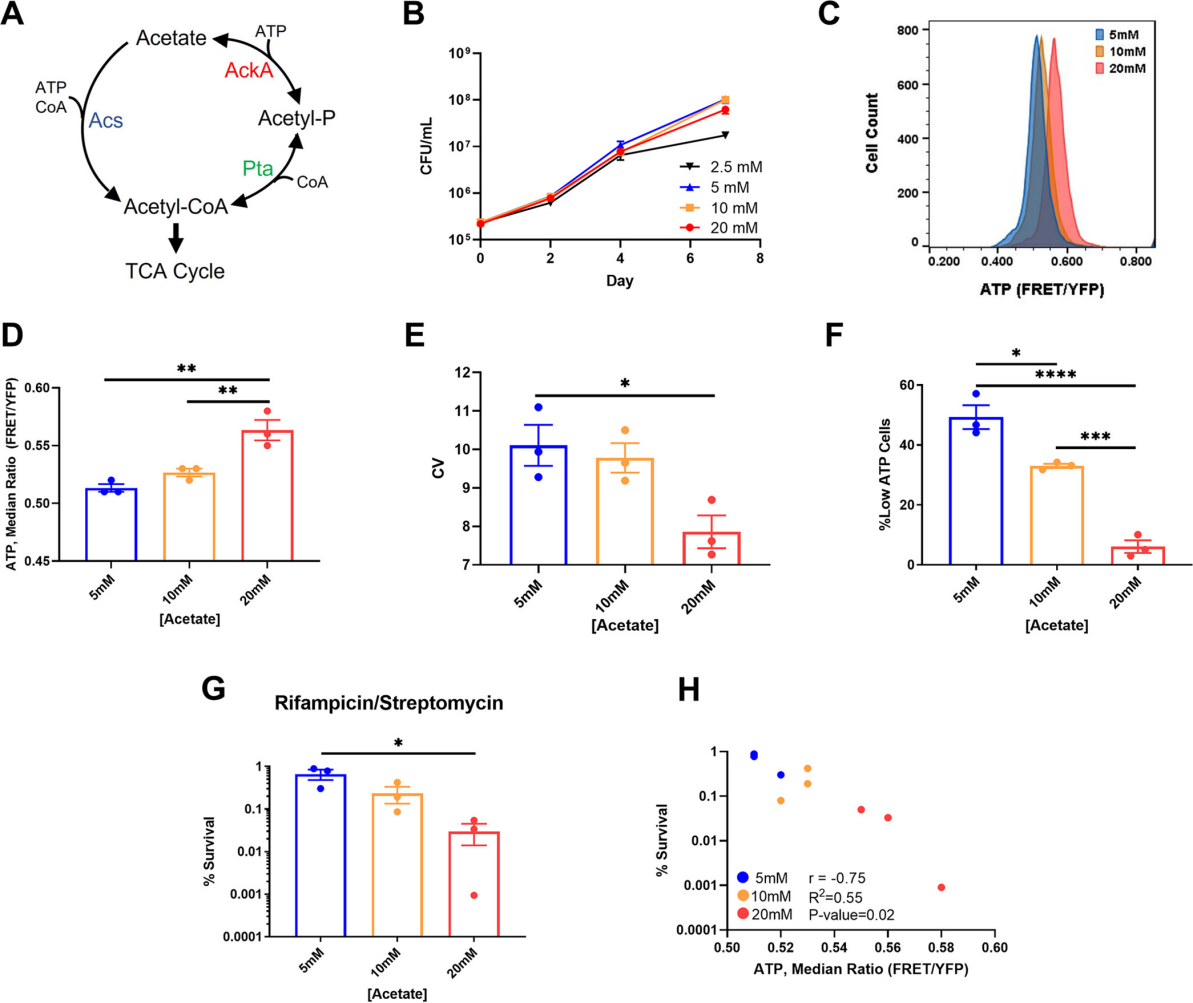
Limiting acetate increases noise in ATP levels and persisters. (A) Schematic of acetate catabolism genes in M. tuberculosis. Acetate is converted to acetyl-CoA by Acs in a single step reaction or by AckA and Pta in a two-step reaction. (B) Growth curve of M. tuberculosis in minimal medium with various concentrations of acetate as the sole carbon source. (C) Representative example of flow cytometry analysis of M. tuberculosis expressing ATeam1.03^YEMK^. M. tuberculosis was grown in minimal medium with the indicated concentrations of acetate for 1 week before being analyzed. (D) Quantification of median FRET/YFP ratio generated by Ateam1.03^YEMK^ in panel C. (E) Quantification of coefficient of variation (CV) of the FRET/YFP ratio in panel C. (F) Quantification of low-ATP cells, defined as events falling below a gate set at a FRET/YFP ratio 1 standard deviation below the median ratio in the 20 mM sample, the sample with the highest median FRET/YFP ratio. (G) Survival of M. tuberculosis grown at the indicated concentrations of acetate after being challenged with rifampicin (10 μg/mL) plus streptomycin (10 μg/mL) for 7 days. (H) Correlation analysis of survival and median FRET/YFP ratio of populations. *, *P* < 0.05; **, *P* < 0.01; ***, *P* < 0.001; ****, *P* < 0.0001. Data are representative of at least three biological replicates. Significance determined by one-way analysis of variance (ANOVA) with Tukey’s posttest.

We previously showed that cells expressing lower levels of TCA cycle intermediates are more tolerant of antibiotics (25–27), suggesting that natural variation in gene expression could drive persister formation. Variation, or noise, in the level of expression of any one among numerous enzymes participating in energy production could lead to the observed subset of cells within the population entering a low-ATP state. In order to identify such “noisy” components, we used a simple growth medium, where the number of enzymes contributing to energy production is minimized. M. tuberculosis grows well in a minimal medium with acetate as a single carbon source, with a doubling time of ~21 h, similar to its doubling time in rich media (18 to 20 h). M. tuberculosis uses two short metabolic pathways that lead from acetate to acetyl coenzyme A (acetyl-CoA), which then enters the TCA cycle (Fig. 3A). Acetate can be converted to acetyl-CoA either in a single step by acetyl-coenzyme A synthase (Acs) or in a two-step pathway consisting of the acetate kinase AckA, producing acetyl phosphate, and the phosphotransacetylase Pta.

We reasoned that consequences of an enzyme being expressed at low levels will be more apparent if the substrate is not saturating the pathway. To this end, we first tested growth of M. tuberculosis at different concentrations of acetate, aiming to identify a minimal level at which growth is not affected. Growth rates were similar in the presence of 20, 10, and 5 mM acetate and dropped at 2.5 mM acetate (Fig. 3B). Importantly, this indicates that acetate does not become limiting over the 7-day outgrowth at the 5, 10, and 20 mM concentrations. If acetate were to become limiting (2.5 mM), any increase in tolerance cannot be solely attributable to noise in gene expression. The level of ATP dropped in the order 20, 10, and 5 mM acetate (Fig. 3C and D). We observed a similar trend with lactate as a single carbon source (see Fig. S1A to C in the supplemental material). We also observed an increase in the cell-to-cell variance in ATP (Fig. 3E). For this, the coefficient of variation (CV) was derived from the FRET/YFP distributions generated via single-cell FACS analysis (Fig. 3C). The CV quantifies variance by dividing the standard deviation (σ) of the FRET/YFP ratio of cells in a population by the mean (μ) FRET/YFP ratio of the population (σ/μ). Variation in ATP levels among cells decreased in the order 5, 10, and 20 mM acetate (Fig. 3E and F). Again, we observed a similar phenotype in a medium with lactate (Fig. S1D and E). The combined effects of a decrease in median ATP and an increase in cell-to-cell variance in ATP suggest that more cells will shift to the low-ATP tail of the distribution in a medium with lower acetate or lactate. As a result, more persisters are expected to form when the carbon source is lowered. Indeed, a population growing in 5 mM acetate, or 10 mM lactate, produced about 10-fold more persisters than cells in a 20 mM sample (Fig. 3G; Fig. S1F). As was the case with bedaquiline pretreatment (Fig. 1F), survival is negatively correlated with median ATP of the population (Fig. 3H). By simply decreasing the amount of carbon available to a population, while maintaining growth rate, we significantly increased the number of persisters formed in the population. These results suggest that aggregate noise in metabolic pathways affects population ATP dynamics and persister levels.

**FIG 4:**
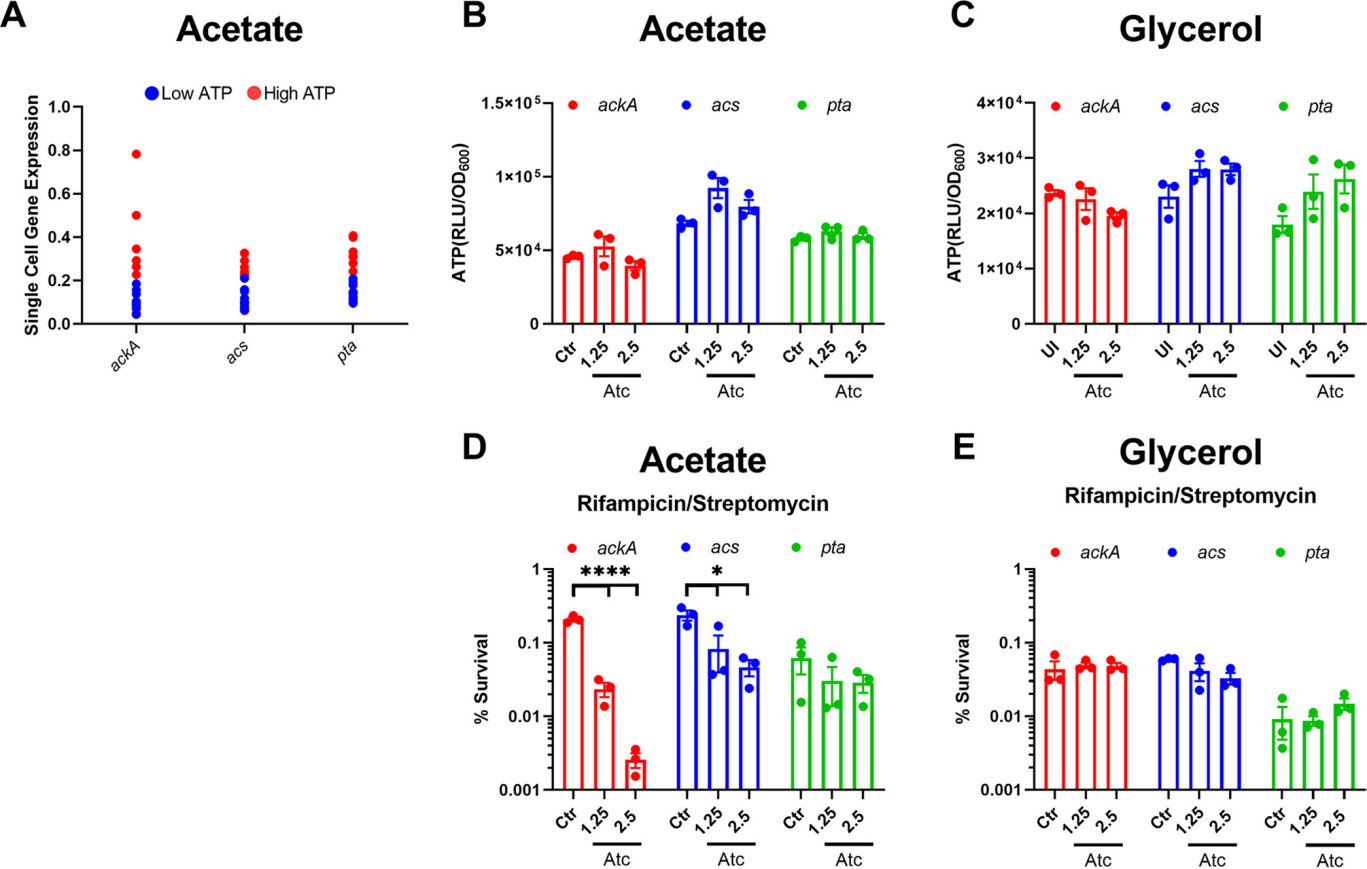
Noise quenching in *ackA* expression reduces drug-tolerant persisters. (A) Single-cell expression analysis of low- and high-ATP cells sorted from a minimal medium with 2.5 mM acetate as the carbon source. M. tuberculosis expressing ATeam1.03^YEMK^ was analyzed via FACS, and a single low- or high-ATP cell was dispensed into a well of a 96-well qPCR plate. Low- and high-ATP cells were gated as in Fig. 2A. Normalized expression of the indicated genes was determined. Expression was normalized to the *C_T_* value determined from the origin of replication of pND235-YEMK (plasmid expressing ATeam1.03^YEMK^). (B and C) ATP in a population of M. tuberculosis overexpressing *acs*, *ackA*, or *pta* in minimal medium with 2.5 mM acetate (B) or 0.01% glycerol (C) as the sole carbon source. ATP was measured by luciferase after 1 week of growth, with or without induction with ATc. Luminescence is normalized to the OD_600_ of the culture. (D and E) Survival of M. tuberculosis expressing *acs*, *ackA*, or *pta* under the control of a tetracycline-inducible promoter. Cultures were grown in minimal medium with (D) 2.5 mM acetate or (E) 0.01% glycerol as the sole carbon source for 7 days. The cultures were left uninduced (Ctr) or induced with ATc (concentrations are in nanograms per milliliter). Cultures were then challenged with rifampicin (10 μg/mL) plus streptomycin (10 μg/mL) for 7 days. The numbers of CFU per milliliter were determined before antibiotic treatment and after 7 days of treatment. *, *P* < 0.05; ****, *P* < 0.0001. Data are representative of at least three biological replicates. Significance was determined by one-way ANOVA with Tukey’s posttest.

While Fig. 3 illustrates the effects of cumulative noise in the metabolic pathways used to catabolize acetate, we next asked if noise in a specific enzyme can generate low-ATP persister cells. To this end, we used single-cell reverse transcription-quantitative PCR (RT-qPCR) recently developed for mycobacteria (18) to directly link ATP concentration to the level of expression of enzymes in the acetate metabolic pathways. For this, we used cells growing at a suboptimal level of acetate (2.5 mM) in order to maximize possible cell-to-cell differences in ATP. Additionally, the effects of variation of a specific enzyme may be masked when acetate is not limiting (≥5 mM acetate). Twelve individual low- and high-ATP cells were sorted into a microtiter plate, and transcripts of the acetate metabolism genes *acs*, *ackA*, and *pta* were measured by RT-qPCR from each low- and high-ATP cell. The low-ATP cells had lower levels of transcripts, while the high-ATP cells produced more transcripts (Fig. 4A). Compared to *acs* and *pta*, there was a particularly large difference in the expression levels of *ackA*, coding for acetate kinase, between low- and high-ATP cells. Additionally, there was a larger variance in expression in *ackA* compared to *acs* and *pta* within the high-ATP cells, suggesting that *ackA* is the source of noise under these growth conditions.

We reasoned that quenching noise in a particular enzyme will diminish persisters if it is indeed responsible for their formation. To test this, we cloned *ackA*, *acs*, and *pta* genes into the plasmid pTetSG under the control of a tetracycline-inducible promoter. By inducing expression from this strong promoter and uniformly increasing expression in the population, we can eliminate the effects of noisy expression from the gene’s endogenous promoter. Cultures were grown in minimal medium with acetate in the presence of anhydrotetracycline (ATc). Induction of expression was confirmed in bulk cultures via RT-qPCR (Fig. S2) and did not affect growth of these strains (Fig. S3). Regardless of carbon source, no change in ATP was seen in the bulk population induced with ATc (Fig. 4B and C). This is to be expected, since only persisters that express smaller amounts of energy-producing components will be sensitive to a decrease in substrate concentration. Changes in the metabolic state of this small subpopulation will not affect bulk ATP measurement. Strikingly, induction of *ackA* resulted in a 100-fold decrease in persisters tolerant of killing by Rif/Strep (Fig. 4D). There was a notable but considerably smaller decrease in persisters upon overexpression of *acs* and no change in cells overexpressing *pta*. Overexpression of *ackA* could result in the accumulation of the toxic pathway intermediate acetyl-phosphate, which could have explained the decrease in survival observed. However, no decrease in survival was observed when *ackA* was overexpressed in medium containing 5 or 10 mM acetate (Fig. S4). Because acetyl-phosphate would accumulate at all concentrations, it is not responsible for the decrease in survival observed at 2.5 mM acetate. Importantly, survival was unaffected when these genes were overexpressed in minimal medium with suboptimal glycerol (0.01%) (Fig. S5) as the sole carbon source (Fig. 4E). These results suggest that *ackA* is the main noise generator in the acetate metabolic pathway and acts as a persister gene.

## DISCUSSION

Studies of antibiotic tolerance in M. tuberculosis and persister cells in other bacterial species have proceeded along parallel lines, with little cross talk. There is a considerable body of evidence showing that metabolic downshift by a variety of means and mechanisms leads to antibiotic tolerance in different bacterial species (32, 33). Of particular relevance to tuberculosis is the finding that establishing dormancy in M. tuberculosis under hypoxia involves a metabolic switch to synthesis of triglycerides, diminishing the available energy resources for other biosynthetic functions and contributing to antibiotic tolerance (16). Our recent work established that both E. coli and S. aureus persisters are low-ATP cells (26, 34). Consistent with this, we show that M. tuberculosis persisters are also low-ATP cells (Fig. 1 and 2). Additionally, as with E. coli persisters (28), M. tuberculosis persisters are stochastically formed during normal growth (Fig. 2). These data suggest that, as with other bacteria, a low-energy state underlies persister formation in M. tuberculosis.

In S. aureus and E. coli, sorting of cells with low expression of Krebs cycle enzymes enriches for persisters (26, 27, 34). This suggests that noise in expression of energy-generating pathways can result in a low-ATP state and persister formation. To examine this relationship more directly, we made use of simple medium with acetate as the sole carbon source. We reasoned that when a substrate is limiting, noise in energy-generating pathways will become more pronounced and lead to increases in low-ATP cells and persisters. At a minimal concentration of acetate that does not yet diminish growth, ATP level drops, with a concomitant increase in persisters (Fig. 3 and Fig. S1). To monitor ATP and noise in energy-generating components in the same cell, we employed single-cell transcription analysis of cells sorted based on their ATP state determined by the specific fluorescent reporter ATeam1.03^YEMK^. This demonstrated considerable noise in the expression of the acetate kinase AckA in M. tuberculosis cells when acetate was the sole carbon source (Fig. 4A). Further, by quenching noise through overexpression of AckA, we were able to dramatically decrease persisters (Fig. 4D), identifying AckA as the principal noise generator under these conditions.

Apart from a general low-energy mechanism, several specialized mechanisms in bacteria that operate under particular conditions have been described. In E. coli, DNA damage by fluoroquinolone antibiotics induces the SOS response, leading to expression of the TisB toxin, which forms an ion channel in the membrane, decreases the proton motive force (PMF) and ATP, and is primarily responsible for persister formation under these conditions (35–37). In E. coli, a gain-of-function *hipA7* (high persister) mutation in the HipA toxin produces *hip* (high persister) mutants (38, 39) that are found in patients treated for urinary tract infection (40). Whether specialized mechanisms of persister formation exist in M. tuberculosis is an important open question. This is especially significant since mutations leading to increased tolerance have been shown to favor development of classical resistance in E. coli and S. aureus (41–44). Whether *hip* mutations in M. tuberculosis favor selection of resistant mutants remains to be established.

A number of studies have linked persisters to disease, starting with the isolation of Pseudomonas aeruginosa *hip* mutants from patients with cystic fibrosis undergoing lengthy antibiotic therapy (45, 46). *hip* mutants have been identified in clinical isolates of M. tuberculosis as well (17). In the case of Salmonella, entrance of cells into macrophages results in a dramatic increase in persisters (47). M. tuberculosis similarly colonizes macrophages, upon which antibiotic tolerance of the bulk population increases (48, 49). In S. aureus, a decrease in ATP and antibiotic tolerance *in vivo* can result from inhibition of respiration by compounds originating from a coinfection with P. aeruginosa and by reactive oxygen species (ROS) produced by macrophages (50–52).

In M. tuberculosis, there are two different paths leading to quiescence, and both are likely responsible for the lengthy antibiotic therapy required to treat tuberculosis. A population entering dormancy in response to external stressors such as hypoxia and stochastically formed persisters appear to share the same basic mechanism of antibiotic tolerance, i.e., a low energy state. This suggests that a therapeutic approach against dormant cells will be effective irrespective of which path they used to enter dormancy. However, while we have a sizable and growing (albeit slowly) arsenal of antibiotics that act against regular cells, discovery of antipersister compounds is still in its infancy (53), with but a few examples of such compounds (54–57). An alternative approach is pulse-dosing with conventional antibiotics, which has been described for eradicating a biofilm formed by S. aureus *in vitro* (58). Combatting dormancy will require novel types of compounds and approaches.

**FIG 5:**
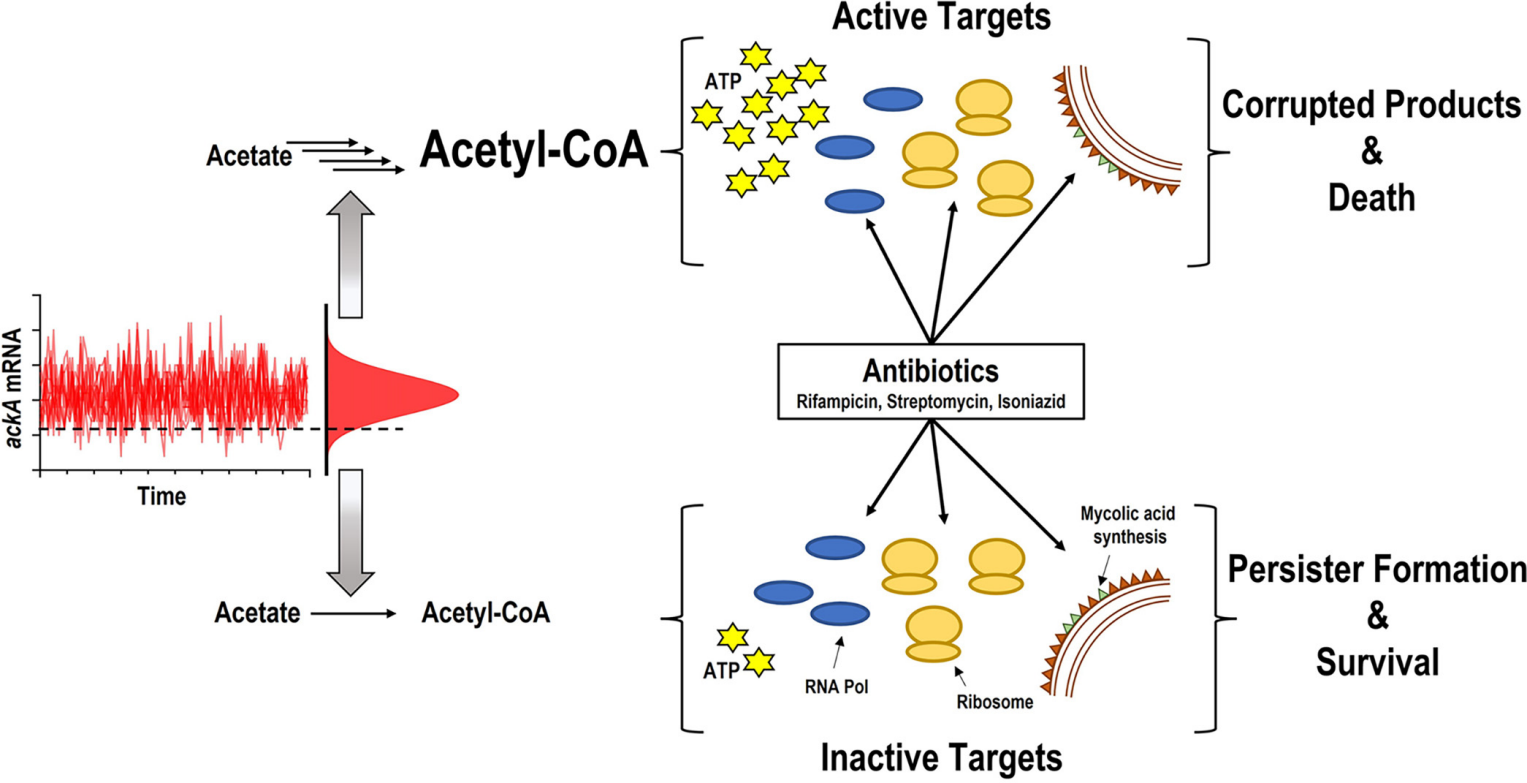
Model of persister cell formation. When M. tuberculosis is grown in minimal medium with acetate, the acetate kinase AckA represents a bottleneck in the energy-producing pathway. In the majority of cells, AckA is not limiting and allows the efficient catabolism of acetate. This translates into higher levels of ATP and active targets such as RNA polymerase, the ribosome, and mycolic acid synthesis. Antibiotics corrupt the targets, resulting in cell death. Noise in transcription causes stochastic decreases in AckA. This leads to a decrease in ATP and inactive targets, creating a multidrug-tolerant persister cell.

Our findings suggest a general model for persister formation in bacteria, which is shared by M. tuberculosis (Fig. 5). Noise in the expression of an energy-generating component, such as *ackA*, results in rare cells that have low levels of ATP. This in turn will decrease the activity of targets, preventing antibiotics from corrupting them. The nature of the principal noise generator will depend on which metabolic pathway is dominant under given growth conditions. From this perspective, there will be many “persister genes” in M. tuberculosis and other bacteria. Noise quenching that we describe in this study provides a direct means to test the involvement of a given gene in persister formation. This approach should also be applicable *in vivo*, where tetracycline-inducible gene expression has been used. Notably, noise quenching by overexpression provides a simple approach to quantitatively determine the relative input of any gene into persister formation.

## MATERIALS AND METHODS

### Bacterial strains and media.

The M. tuberculosis strain used for all experiments was H37Rv mc^2^6020 (59). M. tuberculosis was grown in Difco 7H9 supplemented with 10% oleic acid-albumin-dextrose-catalase (OADC), 0.5% glycerol, lysine (80 μg/mL), pantothenate (24 μg/mL), and 0.05% tyloxapol. Cultures were grown in 30-mL square PETG (polyethylene terephthalate copolyester, glycol modified) bottles. For CFU enumeration M. tuberculosis was plated on Difco 7H10 supplemented with 10% OADC, 0.5% glycerol, lysine (80 μg/mL), and pantothenate (24 μg/mL). To make minimal medium, 0.5 g asparagine, 1 g NH_2_PO_4_, 2.5 g Na_2_HPO_4_, 50 mg ferric ammonium citrate, 0.5 g MgSO_4_, 0.5 mg CaCl_2_, and 0.1 mg ZnSO_4_ were dissolved in 1 L of water. Lysine (80 μg/mL), pantothenate (24 μg/mL), and tyloxapol (0.05%) were added, and the medium was filter sterilized. Carbon sources were added to the medium at the indicated concentrations prior to experiment. For analysis of single-cell ATP, plasmid pND235-YEMK was transformed into M. tuberculosis mc^2^6020 using a Bio-Rad Gene Pulser Xcell set at 2.5 kV, 25 μF, and 1,000 Ω and selected on 7H10 complete medium supplemented with 40 μg/mL kanamycin. For construction of overexpression strains, genes were amplified using the primers listed in Table S1 and cloned into plasmid pTetSG (60). Plasmids were transformed into M. tuberculosis and selected on 7H10 complete medium supplemented with 40 μg/mL kanamycin.

### Growth measurements.

For analysis of growth in minimal medium with single carbon sources, M. tuberculosis was grown in 7H9 complete medium, washed twice in PBS, then resuspended in minimal medium with the indicated carbon source to an optical density at 600 nm (OD_600_) of ~0.01 at a volume of 10 mL. The cultures were grown for a week with shaking at 37°C. Samples were plated for CFU enumeration at days 0, 2, 4, and 7.

### Antibiotic survival assay.

For analysis of bedaquiline effects on survival, cultures were challenged in exponential phase when the OD_600_ was ~0.8 with 12.5 μg/mL (100× MIC). For analysis of survival in exponential and stationary phases, cultures were challenged when the OD_600_ was ~0.8 (exponential phase) or ~1.8 to 2 (stationary phase). For analysis of survival in minimal medium as well as overexpression analysis in minimal medium, cultures were grown for 7 days under the indicated conditions and then challenged. In all cases, an aliquot was removed before treatment, serially diluted, and plated for day 0 CFU enumeration. Cultures were treated with the indicated antibiotic(s) for 7 days, after which an aliquot was removed, washed once in phosphate-buffered saline (PBS), serially diluted and plated for CFU enumeration. Percent survival was calculated as follows: (CFU day 7/CFU day 0) × 100. For analysis of survival in bulk cultures, antibiotics used were rifampicin (10 μg/mL) plus streptomycin (10 μg/mL) or isoniazid (20 μg/mL). Sorted cells were treated with rifampicin (1 μg/mL) or streptomycin (2 μg/mL).

### ATP quantification.

Prior to antibiotic treatment, 500-μL aliquots of cultures were washed once and resuspended in 500 μL PBS. Two 100-μL aliquots of washed samples were transferred to a clear-bottom 96-well plate, and the OD_600_ was measured on a Biotek Synergy H1 plate reader in technical duplicate for each sample. Next, a BacTiter Glo kit (Promega, Madison, WI, USA) was used to measure intracellular ATP. Briefly, two 100-μL aliquots of the sample were transferred to a white 96-well plate, and 100 μL BacTiter Glo reagent was added. The cell suspension was mixed to lyse cells and incubated for 5 min at room temperature. Bioluminescence was measured on a Biotek Synergy H1 plate reader. Bioluminescence values (in relative light units [RLU]) were normalized to OD_600_.

### Flow cytometry and fluorescence-activated cell sorting.

Single-cell ATP was analyzed using M. tuberculosis mc^2^6020 expressing pND235-YEMK. This plasmid encodes a FRET-based ATP biosensor adapted for use in M. tuberculosis (31). FRET-based fluorescence of single cells was collected on a BD FACS Aria II flow cytometer (BD Biosciences, San Jose, CA, USA) with a 70-μm nozzle. Fluorescence was collected for YFP emission at two separate laser excitations with band pass filters optimized for YFP, excitation at 445 nm (CFP_ex_→YFP_em_) (FRET) and excitation at 488 nm (YFP_ex_→YFP_em_). Single-cell-normalized ATP is expressed as the FRET/YFP ratio [(CFP_ex_→YFP_em_)/(YFP_ex_→YFP_em_)]. For flow cytometry analysis of cultures expressing pND235-YEMK, a minimum of 20,000 events were collected. The events were gated for size (forward scatter area [FSC-A] and side scatter area [SSC-A]) and YFP positivity, and finally, the ratiometric signal of ATP (FRET/YFP) was determined. Aliquots of the cultures were directly analyzed on the flow cytometer under the experimental medium conditions. All analysis was conducted in FlowJo (BD).

For survival sorting experiments, a liquid culture of M. tuberculosis expressing pND235-YEMK was grown to stationary phase and then diluted 1:100 in fresh 7H9 medium. The culture was grown to late stationary phase (~2 weeks), diluted 1:20 in PBS, and loaded onto the BD FACS Aria II. To sort based on the normalized ratiometric signal from pND235-YEMK, the ratio feature of the FACS Diva software was enabled to calculate FRET/YFP signal in real time. Events were first gated for size (FSC-A and SSC-A), and YFP positivity, followed by analysis of single cells as a dot plot of FRET/YFP versus YFP signal. “Low ATP” and “High ATP” gates were set to 2% of the total population. YFP^+^ cells were gated as any cell expressing YFP above background levels. A total of 5,000 low-ATP, high-ATP, or YFP^+^ cells were sorted directly into 1 mL 7H9 liquid medium containing either rifampicin (1 μg/mL) or streptomycin (2 μg/mL). The sample was immediately serially diluted and plated on 7H10 to determine the number of CFU per milliliter on day 0. The sample was then plated to determine the number of CFU per milliliter on days 1, 2, and 3 posttreatment. At all time points, a 95-μL aliquot of each sample was removed and added to 5 μL 1% activated charcoal (final concentration of activated charcoal is 0.05%) before being serially diluted to limit the effects of the antibiotics in the medium on CFU determination. Two technical replicates were collected per experiment, reversing the order of sample collection between replicates to limit effects of collection timing bias. The experiment was repeated a minimum of three times.

### Bulk RT-qPCR.

M. tuberculosis cultures were pelleted and resuspended in 1 mL TRIzol (Thermo Fisher, Waltham, MA, USA) followed by mechanical disruption to lyse cells. RNA was extracted with chloroform, precipitated with 100% isopropanol, and washed with 70% ethanol. Purified RNA was treated with Turbo DNA-free (Invitrogen) for 1 h. SuperScript IV Vilo master mix (Invitrogen, Waltham, MA, USA) was used to synthesize cDNA according to the manufacturer’s instructions. Expression of each gene of interest was conducted using Bio-Rad SsoAdvanced universal SYBR green supermix on a Bio-Rad CFX96 system.

### Single-cell RT-qPCR.

The protocol for single cell RT-qPCR was based on a recently developed method (18). Cultures expressing the integrating plasmid pND235-YEMK growing in minimal medium with 2.5 mM acetate as the sole carbon source were grown to stationary phase. Low-ATP, high-ATP, and YFP^+^ cells were sorted based on the criteria described above. Single low-ATP, high-ATP, and YFP^+^ cells were sorted directly into individual wells of a 96-well Bio-Rad (Hercules, CA, USA) PCR plate containing 2 μL lysis solution comprising 10% NP-40, SuperScript IV Vilo master mix (Invitrogen, Waltham, MA, USA), T4 Gene32 (New England Biolabs [NEB], Ipswich, MA, USA), SUPERase RNase Inhibitor (Invitrogen), and 10 pM RNA spike in the control. Firefly luciferase (FLuc) RNA served as the spike in the control and was generated using a HiScribe T7 Quick RNA synthesis kit (NEB, Ipswich, MA, USA) using linearized plasmid DNA carrying the FLuc gene as the template. A total of 16 low-ATP, high-ATP, and YFP^+^ cells were sorted per experiment. After sorting, the cells were lysed by first flash freezing in liquid nitrogen and then placing the plate at −80°C for 1 h, followed by thawing at room temperature. The plate was then transferred to a thermocycler, and cDNA was generated following cycling conditions in the manufacturer’s protocol. Following cDNA synthesis, 25 cycles of preamplification was conducted using gene-specific primers. Excess primer was then removed via incubation with exonuclease I (Thermo Fisher) for 1 h at 37C. One-tenth (2 μL) of the preamplified cDNA was then used to assess the single-cell expression of each gene of interest using Bio-Rad SsoAdvanced universal SYBR green supermix on a Bio-Rad CFX96 system. Amplification of the E. coli origin of replication (oriE) from pND235-YEMK served as the lysis control. Wells that generated no amplification or a threshold cycle (*C_T_*) of >40 for oriE were considered failed lysis and removed from analysis. For all other genes, amplification *C_T_* values of >40 were removed from analysis. *C_T_* values of genes were corrected for the difference between FLuc *C_T_* in each well and the median FLuc *C_T_*. Finally, expression of each gene was normalized to oriE amplification in each well, as it is assumed that oriE exists as a single copy. All primers used for single-cell RT-qPCR analysis can be found in Table S1.

### Statistics.

Details of statistical analysis conducted can be found in figure legends, including tests used to determine significance. Error is represented as standard error of the mean unless otherwise noted. All statistical analysis was conducted using GraphPad Prism V9.
