# Supplementary material for: Noise in a Metabolic Pathway Leads to Persister Formation in Mycobacterium tuberculosis
Source: Microbiol Spectr. 2022 Oct 4;10(5):e02948-22. doi: 10.1128/spectrum.02948-22 (PMC9602276; doi:10.1128/spectrum.02948-22)
Supplement: Supplemental file 1 — Supplemental material. Download spectrum.02948-22-s0001.pdf, PDF file, 0.5 MB [file spectrum.02948-22-s0001.pdf]

Noise in a metabolic pathway leads to persister formation in *Mycobacterium tuberculosis*

Jeffrey Quigley, Kim Lewis*

Antimicrobial Discovery Center, Department of Biology, Northeastern University, Boston, MA, USA
*Corresponding author: Kim Lewis, k.lewis@northeastern.edu

Supplementary Material

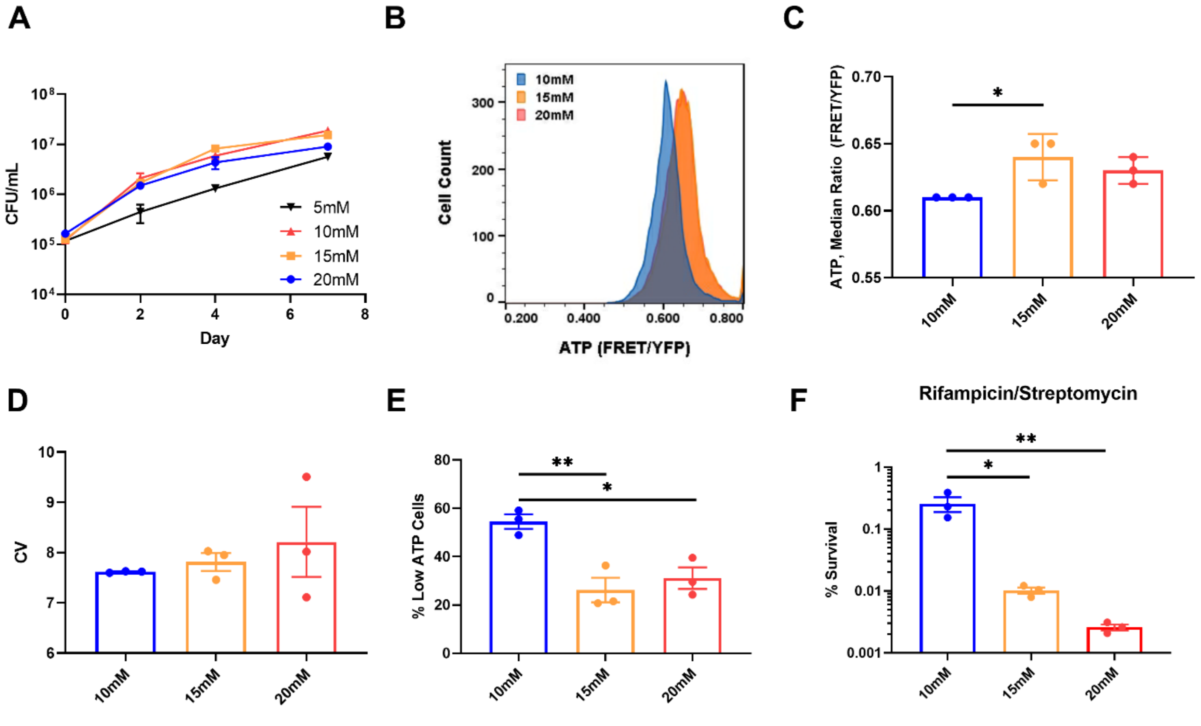

Fig S1 Limiting lactate decreases ATP levels, and increases persisters
(A) Growth curve of *M. tuberculosis* in minimal media with varying concentrations of lactate as the sole carbon source. (B) Representative example of flow cytometry analysis of *M. tuberculosis* expressing ATeam1.03^{YEMK}. *M. tuberculosis* was grown in minimal media with the indicated concentrations of lactate for one week before being analyzed. (C) Quantification of median FRET/YFP ratio generated by ATeam1.03^{YEMK} in (B). (D) Quantification of co-efficient of variation (CV) of FRET/YFP ratio signal in (B). (E) Quantification of "Low ATP Cells" defined as events falling below a gate set at FRET/YFP ratio one standard deviation below median ratio in 20 mM sample, the sample with the highest median FRET/YFP ratio. (F) Survival of *M. tuberculosis* grown at indicated concentrations of lactate after being challenged with rifampicin (10 µg/mL) + streptomycin (10 µg/mL) for seven days. P< 0.05, *, P< 0.01, **. Data are representative of at least three biological replicates. Significance was determined by one-way ANOVA with Tukey's post test.

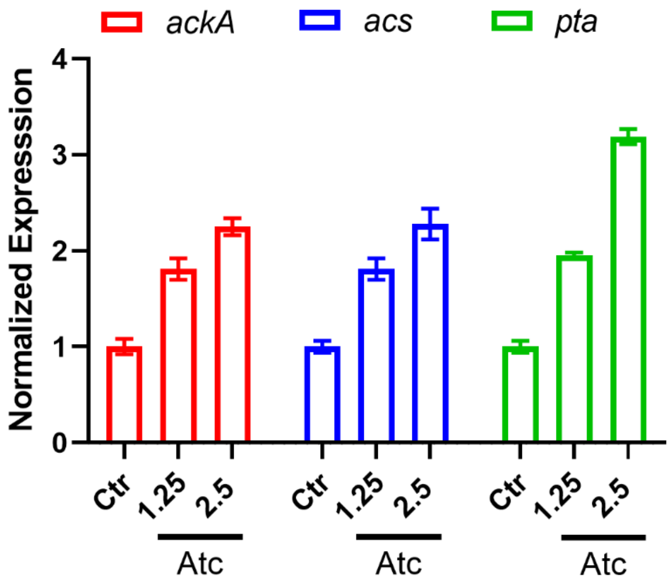

Fig S2 Induction of acetate catabolic genes
Reverse transcription quantitative PCR (RT-qPCR) was used to confirm induction of acetate catabolic genes after induction with anhydrotetracycline (Atc). *M. tuberculosis* expressing ackA, acs, or pta under the control of a tetracycline inducible promoter were grown in minimal media supplemented with 2.5 mM acetate as the sole carbon source for 7 days. Cultures were left uninduced (Ctr) or inducted with Atc at 1.25 or 2.5 ng/mL. At day 7, RNA was purified for qRT-PCR analysis. Expression was normalized to 16s ribosomal RNA. Data are representative of three biological replicates.

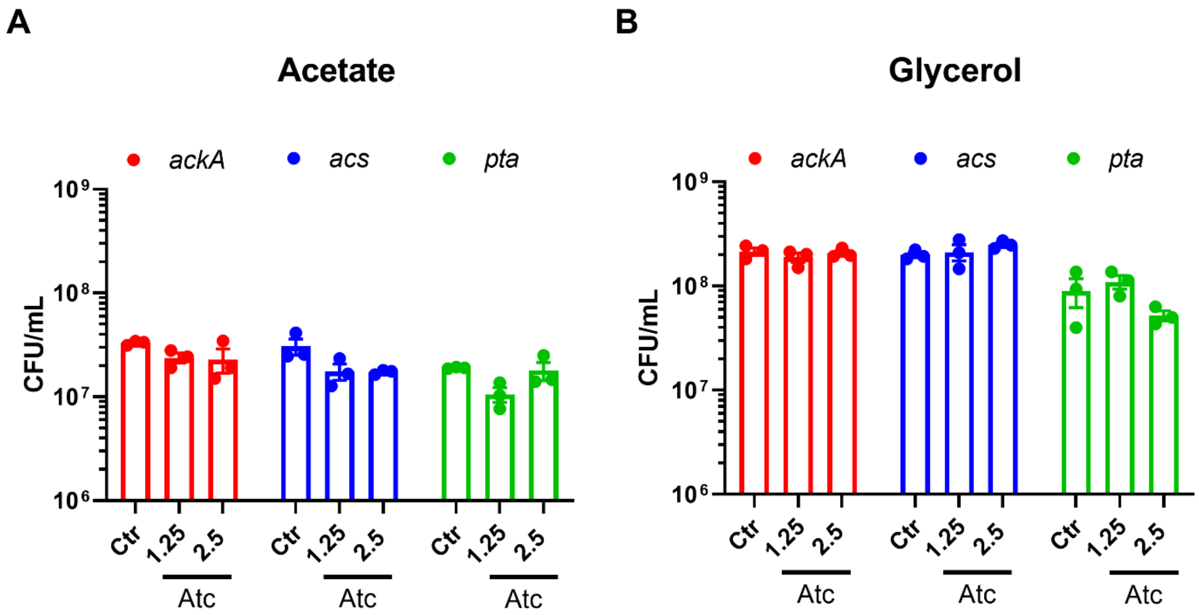

Fig S3 Overexpression does not affect growth of *M. tuberculosis*
Initial CFU/mL of *M. tuberculosis* expressing *acs*, *ackA*, or *pta* under control of a tetracycline inducible promoter. Cultures were grown in minimal media with (A) 2.5 mM acetate or (B) 0.01% glycerol as the sole carbon source for 7 days. The cultures were left uninduced (Ctr) or induced with Atc. Data are representative of three biological replicates.

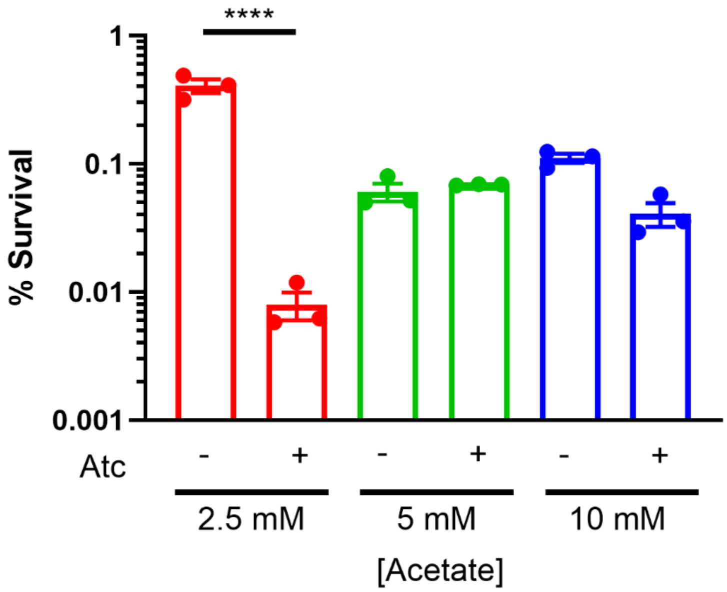

Fig S4 Noise quenching by *ackA* overexpression is only apparent in limiting acetate conditions Survival of *M. tuberculosis* expressing *ackA* under control of a tetracycline inducible promoter. Cultures were grown in minimal media with 2.5, 5, or 10 mM acetate as the sole carbon source for 7 days. The cultures were left uninduced (Ctr) or induced with Atc (ng/mL). Cultures were then challenged with rifampicin (10 µg/mL) + streptomycin (10 µg/mL) for 7 days. CFU/mL were determined before antibiotic treatment and after 7 days of treatment. P< 0.0001, ****. Data are representative of three biological replicates. Significance was determined by one-way ANOVA with Tukey's post test.

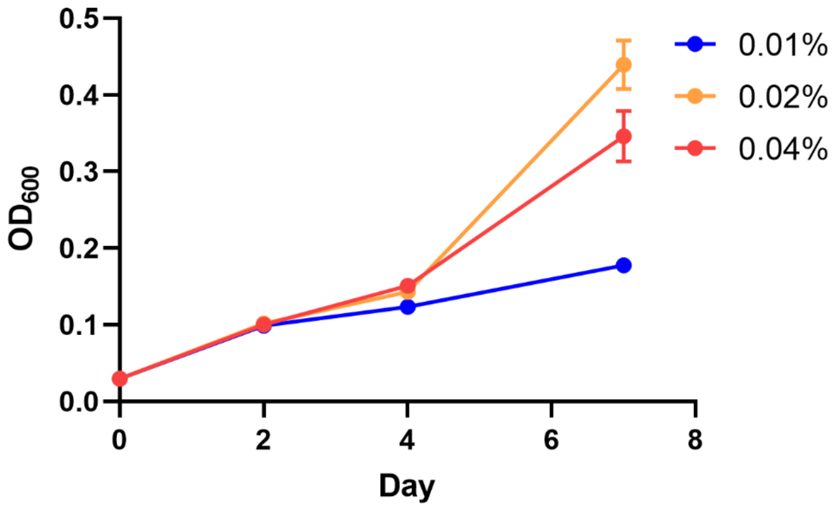

Fig S5 Growth curve of *M. tuberculosis* in glycerol
Growth of *M. tuberculosis* in minimal media with varying concentrations of glycerol as the sole carbon source. Data are representative of three biological replicates.

Table S1: Primers and plasmids used in this study

**<u>Reverse transcription quantitative PCR (RT-qPCR) 5'→3'</u>**
**oriE**
**Forward-GGTTTGTTTGCCGGATCAAG**
**Reverse-TAGCAGAGCGAGGTATGTAG**
**FLuc**
**Forward-CAAAGTGCGTTGCTAGTACC**
**Reverse-GTCTCAGTGAGCCCATATCC**
**ackA**
**Forward-GATGGCATATCCGCCGCTAC**
**Reverse-GTCAGGCCCATCGACGTTTC**
**acs**
**Forward-CAACGTCGCCTACAACTGTG**
**Reverse-TTCGCGGCTTTGGATACCTC**
**pta**
**Forward-CCTGCGATTGCGGTTACCTG**
**Reverse-CAACGCGGTGTCGATCTTGC**
**16s rRNA**
**Forward-GCAACGCGAAGAACCTTACC**
**Reverse-GCGGGACTTAACCCAACATC**
**<u>Overexpression analysis 5'→3'</u>**
**ackA**
**Forward-GGGTTAATTAAGAAGGAGATATACATATGAGTAGCACCGTGCTGGTGATC**
**Reverse-TTTGATATCTCACGCTCGGCGTCCGCCCAGCAC**
**acs**
**Forward-GGGTTAATTAAGAAGGAGATATAATGAGTGAGTCCACCCCCGAAGTC**
**Reverse-AAAGATATCCTACTTGGCGGCCCGGATCGCGTC**
**pta**
**Forward-GGGTTAATTAAGAAGGAGATATACATATGGCTGACTCCTCGGCGATCTAC**
**Reverse-GGCTTTAAACTACTCATGGACGCCCTGCGCCTG**
**<u>Plasmids</u>**
**pND235-YEMK, integrating plasmid constitutively expressing Ateam 1.03^{YEMK} FRET based ATP sensor**
**pTetSG-ackA, episomal tetracycline inducible expression plasmid containing *ackA***
**pTetSG-acs, episomal tetracycline inducible expression plasmid containing *acs***
**pTetSG-pta, episomal tetracycline inducible expression plasmid containing *pta***
